# Yield of routine mycobacterial culture of osteoarticular specimens in a tertiary orthopaedic hospital in England, 2017–2022

**DOI:** 10.5194/jbji-9-217-2024

**Published:** 2024-10-17

**Authors:** Tom A. Yates, Olivier Vahesan, Simon Warren, Antonia Scobie

**Affiliations:** 1 Department of Infection, Royal Free Hospital, London, NW3 2QG, UK; 2 UCL Institute of Health Informatics, University College London, London, NW1 2DA, UK; 3 Division of Infection and Immunity, University College London, London, WC1E 6BT, UK; 4 Bone Infection Unit, Royal National Orthopaedic Hospital, Stanmore, HA7 4LP, UK

## Abstract

**Introduction**: At our tertiary orthopaedic centre, mycobacterial cultures are routinely performed on bone and joint samples sent for bacterial culture. **Methods**: From laboratory records, we ascertained the number of mycobacterial cultures performed, the number positive for *Mycobacterium tuberculosis* complex (MTBC) and/or non-tuberculous mycobacteria (NTM), and the characteristics of individuals from whom mycobacteria were isolated. We collected the same data from 100 individuals with negative mycobacterial cultures. **Results**: Excluding sample types that were not bone or joint samples, 6162 mycobacterial cultures were performed between 4 July 2017 and 30 September 2022. A total of 22 patients had MTBC and 6 patients had NTM newly isolated from bone or joint samples placed in mycobacterial culture, while a further 1 patient had both *Mycobacterium tuberculosis* and *Mycobacterium avium* isolated. To identify one new mycobacterial infection of bone or joint (MTBC or NTM) that would not have been detected with routine bacterial cultures alone, 229 (95 % CI of 158–347) mycobacterial cultures were needed. Mycobacterial cultures were much less likely to be positive in samples taken from prosthetic joints. They were more likely to be positive in spinal samples and in samples taken from patients with suspected sarcoma. In patients from whom MTBC had been isolated, granulomatous inflammation was reported in 86 % (18 of 21) of contemporaneous histological specimens. **Conclusions**: Targeted, rather than routine, mycobacterial culture of bone and joint specimens should be considered in settings with a low burden of tuberculosis.

## 1 Background

Overall, the incidence of tuberculosis (TB) in England is low, with a case notification rate of 7.8 per 100 000 per year (2021) (UK Health Security Agency, 2022). Cases are unevenly distributed, with notifications in parts of Leicester and London exceeding 40 per 100 000 per year (UK Health Security Agency, 2022). Of the 4425 cases reported in England in 2021, 189 people had spinal TB (4.3 %) and 89 (2.0 %) had TB affecting other bones or joints (UK Health Security Agency, 2022).

We performed a retrospective analysis of the yield of routine mycobacterial cultures at our institution. The Royal National Orthopaedic Hospital (RNOH) is a tertiary orthopaedic hospital in Greater London. The hospital hosts a regional sarcoma unit. This analysis complements a previously published study which described the characteristics of people with bone and joint TB diagnosed at RNOH (Broderick et al., 2018).

Over the period included in this analysis, RNOH bone and joint samples sent for standard microbiological investigations were routinely pooled and placed into mycobacterial liquid culture in addition to routine bacterial culture. This included not only all patients referred with suspected sarcoma but also all arthroplasty revision cases, whether routine or performed for suspected infection.

## 2 Methods

### 2.1 Population

A list of mycobacterial cultures performed on RNOH patient samples was extracted from a laboratory database. We removed non-bone and non-joint sample types, such as sputum or cerebrospinal fluid. Cases were patients with positive mycobacterial cultures on bone or joint samples. For every patient with mycobacterial cultures positive for *Mycobacterium tuberculosis* complex (MTBC), we randomly selected five control patients who had negative routine mycobacterial cultures performed on bone or joint samples over the same period. Cases and controls were frequency matched by calendar year.

The following details were extracted from medical records for both cases and controls: age, sex, ethnicity, year of sampling, anatomical location sampled, whether prosthetic material was present, referral pathway, whether the same organism was isolated from routine bacterial cultures, and whether concurrent histology demonstrated findings suggestive of mycobacterial infection. For patients with non-tuberculous mycobacteria (NTM) isolated from bone or joint samples, we also captured data on whether the patient was treated for NTM, whether the same species of NTM was isolated upon repeat sampling, whether the patient was immunocompromised, and whether there was a history of traumatic injury.

### 2.2 Laboratory processes

Part of each bone and joint sample sent for routine culture during a single procedure was routinely pooled, placed into BACTEC Mycobacteria Growth Indicator Tube (MGIT) culture (BD, Franklin Lakes, USA), and incubated at 37 °C for 42 d. Positive cultures were sent to the reference laboratory for confirmation, speciation, and susceptibility testing.

### 2.3 Statistical analysis

The analysis presented is primarily descriptive, with medians and ranges presented for continuous variables and binomial confidence intervals calculated for proportions. For sex (male vs. female), sample type (prosthetic joint vs. other), location (spine vs. other), and referral pathway (sarcoma vs. other), univariable logistic regression was used to calculate odds ratios for positive mycobacterial cultures. This was first done for MTBC only and then for MTBC and NTM (combined) with the same controls used in both analyses. The matching variable (calendar year) was included as a categorical variable. We planned to disaggregate our results by ethnicity, but missing data meant that this was not possible.

To estimate the number needed to test (NNT) to diagnose one new mycobacterial bone or joint infection, the total number of mycobacterial cultures performed, after removing sample types that were clearly not from bone or joint, was used as the numerator. NNT was also calculated in subgroups: by sex, sample type, location, and referral pathway. Here, the size of each subgroup was estimated as the product of the proportion of controls with each characteristic and the overall sample size. For the subgroups, conservative confidence intervals were obtained using the ends of the 95 % confidence intervals for the proportion positive and the proportion with each characteristic. NNT was calculated for MTBC and separately for MTBC and NTM (combined). The cost of diagnosing mycobacterial bone and joint infections with routine mycobacterial cultures was estimated by multiplying the NNT by the laboratory cost of performing one mycobacterial culture (GBP 48).

We collected data on when patients diagnosed with MTBC bone and joint infections were referred to RNOH, when samples were taken, and when mycobacterial cultures were flagged as positive. These data were collected to allow a comparison with data on diagnostic delay from 2012 to 2014 (Broderick et al., 2018), an earlier period during which mycobacterial culture of osteoarticular samples was not routine.

Data were provided as Excel files (Microsoft Corporation, Redmond, USA). Logistic regression and the calculation of binomial confidence intervals were undertaken using STATA 13.1 (StataCorp, College Station, USA). The Venn diagram was generated using the eulerr package in R (Larsson, 2021).

## 3 Results

### 3.1 Samples

**Figure 1:**
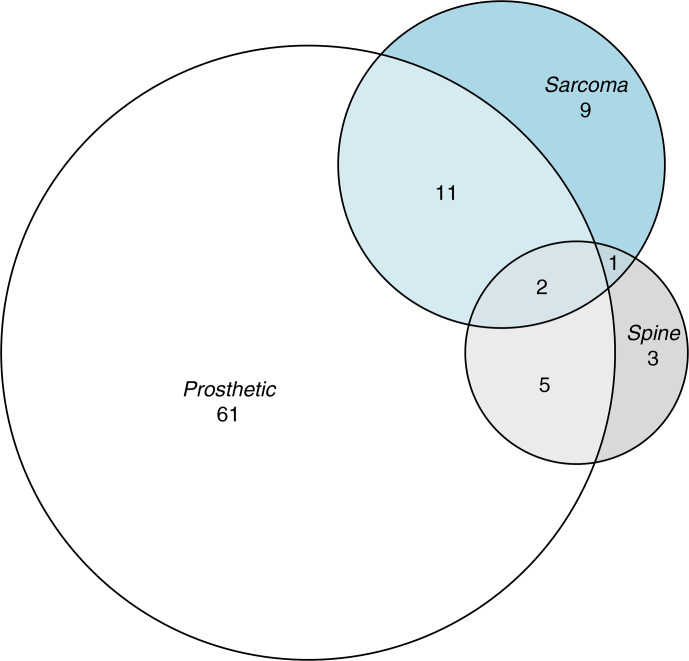
The overlapping characteristics of the 100 frequency-matched controls – 8 controls had none of these characteristics.

We looked at all RNOH samples that underwent mycobacterial culture between 4 July 2017 and 30 September 2022. Excluding sample types that were clearly not from bone or joint, 6162 mycobacterial cultures were left for analysis. From these, we selected all positive cultures and 100 randomly selected controls that were frequency matched by calendar year. The characteristics of cases and controls are given in Table 1 and Fig. 1. Cultures were set up using bone or tissue rather than joint aspirate or other fluid in 21 out of 23 of the samples from which MTBC was isolated (91 %), 4 out of 7 samples from which NTM were isolated (57 %), and 82 out of 100 control samples (82 %).

**Table 1:** The characteristics of patients from whom MTBC was isolated from bone or joint samples, of patients from whom NTM were isolated from bone or joint samples, and of frequency-matched controls whose routine mycobacterial cultures were negative.

	MTBC cases	NTM cases	Controls	Univariable odds	Univariable odds ratio
	( n=23 )^∗^	( n=6 )^∗^	( n=100 )	ratio of MTBC	of MTBC and/or NTM
				(95 % CI)	(95 % CI)
Median age (range)	51 years (15–90)	65 years (39–88)	65 years (8–94)		
Sex					
Female	8 (35 %)	2 (33 %)	57 (57 %)	Reference	Reference
Male	15 (65 %)	4 (67 %)	43 (43 %)	2.5 (1.0–6.4)	2.5 (1.1–6.0)
Sample					
Native bone/joint/disc	20 (87 %)	4 (67 %)	21 (21 %)	Reference	Reference
Prosthetic joint	3 (13 %)	2 (33 %)	79 (79 %)	0.03 (0.01–0.12)	0.04 (0.01–0.14)
Site					
Non-spine	14 (61 %)	6 (100 %)	89 (89 %)	Reference	Reference
Spine	9 (39 %)	0 (0 %)	11 (11 %)	5.8 (1.9–17.4)	4.0 (1.4–11.4)
Referral pathway					
Not sarcoma	6 (26 %)	3 (50 %)	77 (77 %)	Reference	Reference
Sarcoma	17 (74 %)	3 (50 %)	23 (23 %)	10.8 (3.6–32.2)	7.9 (3.1–20.3)

^∗^ One patient from whom both *Mycobacterium tuberculosis* and *Mycobacterium avium* grew in the same sample was counted among the MTBC cases.

### 3.2 *Mycobacterium tuberculosis* complex

A total of 23 samples grew MTBC, including 22 *Mycobacterium tuberculosis* and 1 *Mycobacterium africanum* isolate. The prevalence of MTBC was 0.37 % (95 % CI of 0.24 %–0.56 %). Of samples that were positive for MTBC, 12 (52 %) were taken from a native joint, 6 (26 %) were taken from bone, 3 (13 %) were taken from a prosthetic joint, and 2 (9 %) were taken from an intervertebral disc. Anatomical locations included the foot (4), elbow (3), shoulder (2), thoracic spine (2), lumbar spine (2), sacroiliac joint (2), cervical spine (1), lumbosacral joint (1), sacrum (1), ulnar bone (1), wrist (1), hip (1), femur (1), and a knee (1). Samples that grew MTBC were more likely to be obtained from younger people, men, spinal samples, and patients referred via the sarcoma pathway, but they were much less likely to be obtained from prosthetic joints (Table 1).

### 3.3 Non-tuberculous mycobacteria

NTM were newly isolated from seven patients. There were three rapid growers – two *Mycobacterium abscessus* and one *Mycobacterium chelonae* isolate. There were four slow growers – two *Mycobacterium avium*, one *Mycobacterium malmoense*, and one *Mycobacterium marinum* isolate. Positive NTM cultures were obtained from prosthetic hip joints (2); a prosthetic elbow joint; and samples taken from a native knee, tibia, foot, and wrist.

One of the *Mycobacterium avium* isolates grew in a sample from which *Mycobacterium tuberculosis* had also been isolated. Two of these NTM, both *Mycobacterium abscessus*, grew in routine cultures from the same sample. Therefore, the yield of NTM was 7 isolates in 6162 routine mycobacterial cultures, or 0.11 % (95 % CI of 0.05 %–0.23 %). The yield of additional mycobacteria that would not otherwise have been isolated was 4 in 6162 routine mycobacterial cultures, or 0.06 % (95 % CI of 0.02 %–0.17 %).

Small numbers preclude sensible comment on the characteristics of patients from whom NTM were isolated. Associations between patient characteristics and a combined outcome of positive MTBC and/or NTM cultures were similar to the associations between patient characteristics and MTBC culture positivity (Table 1).

### 3.4 Clinical significance

Contemporaneous histological results were available for 21 out of 23 patients from whom MTBC was isolated from routine bone or joint mycobacterial cultures. A total of 14 (67 %) of these histological samples demonstrated necrotising granulomatous inflammation, 4 (19 %) demonstrated non-necrotising granulomas, 1 (5 %) demonstrated isolated necrosis, and 2 (10 %) showed no histological evidence of tuberculosis.

In two patients, the same species of NTM was recurrently isolated. In the remaining five patients, the clinical significance of the NTM isolates was less clear. Three of the seven samples growing NTM had contemporaneous histological results available, which included the patient from whom both *Mycobacterium tuberculosis* and *Mycobacterium* avium were isolated. None of these three samples revealed granulomatous inflammation; however, all samples demonstrated non-specific chronic inflammation, and features of metallic wear debris from a prosthesis were seen in one case. One further patient with positive *M. marinum* cultures had histology showing necrotising granulomatous inflammation from a sample subsequently obtained from the same joint at another hospital. In five of the seven patients, including the patient from whom *M. tuberculosis* was concurrently isolated, we know clinicians elected to treat for osteoarticular NTM. Five of the seven patients, including the patient from whom *M. tuberculosis* was concurrently isolated, had a history of antecedent traumatic injury or immunocompromise, known risk factors for osteoarticular NTM disease (Brown Elliott and Wallace, 2010).

### 3.5 Number needed to test and the associated costs

Assuming clinical significance, the NNT to diagnose one MTBC bone or joint infection was 268 (95 % CI of 179–423), at a cost to the laboratory of GBP 12 860 (95 % CI of GBP 8579–20 275). The NNT and associated cost varied considerably according to patient characteristics (Table 2) with, for example, an NNT of 65 (GBP 3106) for non-prosthetic joint specimens vs. 1623 (GBP 77 888) in samples taken from prosthetic joints. Adding the four NTM that would not have been diagnosed without routine mycobacterial cultures did not change the yield meaningfully, with an NNT of 229 (95 % CI of 158–347), at a cost to laboratory of GBP 10 955 (95 % CI of GBP 7537–16 612) per newly diagnosed MTBC or NTM bone and joint infection. These figures do not include sampling costs or the costs incurred by the reference laboratory.

### 3.6 Diagnostic delay

In 23 patients diagnosed with osteoarticular TB, the median time from referral to sampling was 22 d (range of 2–53 d), with one patient, initially managed in the private sector, sampled prior to referral. Many of these were urgent referrals for suspected sarcoma. Median time to culture positivity was 14 d (range of 5–124 d), with three isolates only growing following extended incubation. Median time from referral to the cultures flagging positive was 44 d (range of 8–136 d).

**Table 2:** The number needed to test and the associated laboratory costs to isolate one MTBC infection using routine mycobacterial culture of bone or joint specimens.

	Cases	Proportion of	Estimated	NNT	Cost (in GBP) per
		controls	denominator	(CI^∗^)	MTBC isolated (CI^∗^)
Sex					
Female	8	0.57	3512	440 (183–1193)	21 075 (8771–57 242)
Male	15	0.43	2650	177 (83–391)	8479 (3966–18 765)
Sample					
Native bone/joint/disc	20	0.21	1294	65 (27–153)	3106 (1294–7330)
Prosthetic joint	3	0.79	4868	1623 (491–8616)	77 888 (23 529–413 522)
Site					
Non-spine	14	0.89	5484	392 (214–760)	18 803 (10 229–36 456)
Spine	9	0.11	678	76 (21–282)	3616 (974–13 530)
Referral pathway					
Not-sarcoma	6	0.77	4745	791 (319–2374)	37 958 (15 300–113 921)
Sarcoma	17	0.23	1417	84 (35–203)	4002 (1651–9698)
Total	23	1.00	6162	268 (179–423)	12 860 (8579–20 275)

^∗^ Conservative confidence intervals calculated using the ends of the 95 % confidence intervals for both underlying proportions (see Sect. 2).

## 4 Discussion

Routine mycobacterial culture of bone and joint samples rarely yielded MTBC and very rarely yielded NTM, with yield particularly low in samples taken from prosthetic joints. Yield was higher in samples taken from native joints, bone, or intravertebral discs; in spinal samples; and in patients referred with suspected sarcoma. MTBC isolates appeared clinically significant, with granulomatous inflammation usually present in contemporaneous histological specimens. The clinical significance of some NTM isolates was uncertain.

Our finding that yield was higher in men was unsurprising. In England, the male-to-female ratio for diagnosed TB disease is 1.5 (UK Health Security Agency, 2022), consistent with the higher burden observed in adult men globally (Horton et al., 2016; World Health Organisation, 2023). Yield being higher in spinal samples was also unsurprising, with spinal disease comprising 68 % of bone and joint TB diagnosed in the UK and spondylitis, or Pott's disease, being the most common osteoarticular manifestation globally (Marais et al., 2023; UK Health Security Agency, 2022).

Our finding that yield was low in samples taken from prosthetic joints likely reflects both the low incidence of tuberculous prosthetic joint infection and the sampling strategy at RNOH, where cultures are taken routinely at arthroplasty revision, regardless of whether infection is suspected. In studies from the USA, both targeted and routine mycobacterial cultures sent from patients with suspected prosthetic joint infection demonstrate a very low yield (Golden et al., 2022; Tai et al., 2022; Wadey et al., 2010). Note that TB incidence in the USA is 2.6 per 100 000 per year (2022), which is one-third of that in England and nearly 7-fold lower than in London (UK Health Security Agency, 2022; World Health Organisation, 2023). Yield is likely to be somewhat higher in patients from London with suspected prosthetic joint infection.

Our sarcoma finding is important and likely reflects the fact that sarcoma and osteoarticular TB are hard to distinguish clinically (Lex et al., 2019). At another regional sarcoma unit in England, a diagnosis of infection was made in 2.1 % and 0.7 % of referrals for suspected bone and soft-tissue sarcoma, with *Staphylococcus aureus* being the most common organism, followed by *Mycobacterium tuberculosis* (Lex et al., 2019).

Routine mycobacterial culture of bone and joint samples at RNOH was motivated by delays in diagnosing osteoarticular tuberculosis both at our centre (Broderick et al., 2018) and other hospitals in the region (Etti et al., 2021). Osteoarticular TB contributes disproportionately to TB-related morbidity, with early diagnosis and treatment key to preventing joint destruction (Alene et al., 2021; Marais et al., 2023). The characteristics of people with bone and joint TB diagnosed at RNOH in 2012–2014 have been previously described (Broderick et al., 2018). Patients typically presented with pain and joint swelling. Constitutional symptoms were unusual. Patients waited a median of 7 months between the onset of symptoms and referral to RNOH; they then waited a further 2.3 months between referral and the initiation of TB treatment. In the earlier study at RNOH (Broderick et al., 2018), the time from referral to the initiation of TB treatment was greater than 3 and 6 months in 33 % and 10 % of patients respectively. Assuming rapid initiation of therapy once cultures are flagged as positive, two patients in the current study (9 %) may have waited more than 3 months between referral and the initiation of TB treatment. In neither case was a TB PCR performed. No patient waited more than 6 months.

One possible response to these data would be to restrict routine mycobacterial culture of osteoarticular specimens to specific patient groups. For example, 20 of 23 samples from which MTBC was isolated were taken from bone, joint, or discs containing no prosthetic material. Of the three cases in a prosthetic joint, one would have been picked up when the histology was reported, one had histology showing non-specific chronic inflammation only, and one had no histological samples sent. This change would have avoided 79 % of routine mycobacterial cultures, saving GBP 233 663 in direct laboratory costs over this period. If this change were implemented, it may be prudent to retain some specimen for testing, should histology show granulomatous inflammation or the diagnosis remain unclear. Note that, at RNOH, although histology is routinely sent in patients referred with suspected sarcoma, this is not routine during prosthetic joint surgery.

Regarding NTM, the low yield observed in this study is expected. Active surveillance for extrapulmonary NTM disease in various US states estimated an incidence of 1 per 100 000 per year, with osteoarticular disease comprising 4.4 % and 4.2 % of cases (Grigg et al., 2023; Henkle et al., 2017). Single-centre retrospective record reviews in Pakistan and China as well as a review of published cases have suggested that NTM are a rare cause of prosthetic joint infection (Iqbal et al., 2022; Maimaiti et al., 2023). Rapidly growing NTM will often grow in routine bacterial cultures, and adaptations to routine culture methods can improve yield (Tai et al., 2022). In this study, two of three rapidly growing NTM also grew in routine cultures.

There are limitations to this study. First, whilst routine mycobacterial culture of bone or joint specimens was hospital policy over this period, this was not always implemented. One patient had *Mycobacterium fortuitum* isolated from a peri-spinal soft-tissue mass placed in routine culture but not inoculated into MGIT bottles. Such omissions may have marginally inflated our estimates of the yield of routine testing, as cases in which clinical suspicion was high will have been more likely to be tested. However, in our experience, adherence to the policy was high ( >90 %) over this period.

Second, the assumption that removing sample types that were clearly not from bone and joint left a set of osteoarticular samples may not always have been accurate. That said, all of the samples that grew MTBC or NTM and all 100 control samples had been taken from bone or joints. A small number of samples from other sites included in the denominator would tend to bias the estimated yield downward.

Third, limited clinical data were available to us, as many patients attended RNOH for sampling but were managed at local hospitals. We cannot know how many of these mycobacterial bone and joint infections would have been suspected clinically, resulting in the targeted use of mycobacterial cultures. However, previously reported delays in making these diagnoses, in both secondary care and at specialist orthopaedic hospitals, suggest that clinical suspicion is not always sufficient (Broderick et al., 2018; Etti et al., 2021).

Fourth, data on ethnicity were poorly captured, and we did not have data on place of birth. TB incidence in England varies considerably by ethnicity and place of birth, with notifications in UK-born white people of 1.4 per 100 000 per year vs. 99.8 per 100 000 per year among non-UK-born people of Indian ethnicity (2021 data) (UK Health Security Agency, 2022). Targeting mycobacterial cultures according to ethnicity or, better, place of birth may be feasible, but we do not have data to support that approach.

Fifth, TB notifications have halved in England over the last decade (UK Health Security Agency, 2022). This appears to be reflected in the number of diagnoses of osteoarticular TB at RNOH, with 31 cases diagnosed over a previous 2-year period (2012–2014) but only 23 cases diagnosed over the 5 years covered by this audit (2017–2022) (Broderick et al., 2018). The yield of various approaches to testing for mycobacteria will clearly be impacted by local changes in TB incidence.

Sixth, most samples placed in mycobacterial cultures at RNOH comprised bone or tissue. Mycobacterial culture of joint aspirates and other fluids is less sensitive; therefore, the yield from such samples is expected to be lower (Marais et al., 2023).

Seventh, whilst the purpose of our analysis was descriptive (Lesko et al., 2022), it would have been nice to explore whether univariable associations differed within strata of other key explanatory variables. Our data were too sparse to stratify or to fit interaction terms in multivariable analysis. However, the large effect sizes, their clear biological plausibility, and the limited correlation between key explanatory variables (Fig. 1) mean it is unlikely that these univariable associations arose purely due to covariate imbalance between exposed and unexposed groups. Indeed, as outlined above, similar associations have been described in other settings.

Eighth, we have only provided crude estimates of the cost to the local laboratory of detecting a new osteoarticular mycobacterial infection. This is not a cost-effectiveness analysis. In evaluating whether these data can be generalised to other settings, both the local TB incidence and local laboratory costs should be considered.

Finally, RNOH is a tertiary centre seeing high volumes of complex and atypical cases, including sarcoma. The population may, therefore, be enriched with respect to difficult-to-diagnose conditions, including osteoarticular TB, particularly that presenting with atypical history, signs, or investigations. Obvious osteoarticular TB – for example, presenting with multi-level spinal lesions – is likely to have been diagnosed and managed locally. The yield of testing may, therefore, differ from that seen at less specialist centres, and the expected associations between patient characteristics and test positivity may be less marked.

## 5 Conclusions

Routine mycobacterial culture of bone and joint samples has been implemented at RNOH with the intention of reducing the delay in diagnosing osteoarticular TB. Diagnostic delay does appear to have reduced. These data suggest that the yield of MTBC varies considerably by patient characteristics and that NTM are rarely isolated. Our data and the data published by others (Golden et al., 2022; Tai et al., 2022; Wadey et al., 2010) suggest that, in low-burden settings, routine mycobacterial testing of samples taken from prosthetic joints is likely to incur significant cost for little gain. For other samples, the cost-effectiveness of routine mycobacterial cultures likely differs according to patient mix and with changes in local TB epidemiology – more targeted testing will often be warranted. Both this and a previous study at RNOH demonstrated that, in osteoarticular TB, histology usually showed changes consistent with mycobacterial disease and could serve as a prompt to set up mycobacterial cultures (Broderick et al., 2018).

## Data Availability

This project was a service evaluation, and, as such, the dataset comprised the medical and laboratory records of a number of patients. We do not have permission to share these patient-identifiable data, and, even if we had, we would struggle to do so in a way that would prevent individuals being identified.

## Code availability

The STATA code used to perform the regression analyses is available at https://github.com/tayates/rnoh_tb (tayates, 2024).
